# Exploring the relationship between artificial intelligence and resilience in manufacturing industrial chains: mechanisms, effects and empirical evidence

**DOI:** 10.1038/s41598-025-34829-z

**Published:** 2026-01-06

**Authors:** Sirui Liu, Yang Fu, Hanqi Song, Ping Han

**Affiliations:** 1 School of Economics, Harbin University of Commerce, Harbin, 150028 China; 2 Heilongjiang University of Science and Technology, Harbin, 150022 China; 3 School of Accounting, Harbin Finance University, Harbin, 150030 China

**Keywords:** Artificial intelligence, Resilience of manufacturing industrial chains, Regional economic development, Development of data elements, Engineering, Mathematics and computing

## Abstract

Using panel data from 30 Chinese provinces for the period 2012–2023, this study systematically examines the mechanisms, nonlinear characteristics, and spatial heterogeneity of artificial intelligence’s impact on the resilience of manufacturing industrial chains. The results indicate that AI exerts a significant and robust direct positive effect on industrial chain resilience. Furthermore, AI indirectly enhances resilience by promoting regional economic development. The urbanization rate positively moderates this relationship, with a higher urbanization level amplifying AI’s enabling effect. A threshold analysis reveals that the influence of AI exhibits nonlinear characteristics based on the development level of data elements; beyond a certain threshold, its positive effect displays a pattern of “marginal increase.” Heterogeneity analysis shows that AI’s enabling effect varies regionally, being strongest in the east, followed by the west, and least pronounced in the central region. Moreover, this effect intensifies with higher levels of supply chain resilience, suggesting a “Matthew effect” whereby stronger chains benefit more. This study provides theoretical and empirical insights into how digital technologies enhance industrial resilience and offers policy implications for designing differentiated and coordinated AI promotion strategies.

## Introduction

The global industrial chain is currently undergoing profound restructuring and fundamental shifts in technological paradigms. Geopolitical turbulence, sudden external shocks, and increasingly complex supply chain risks pose severe challenges to the stability and sustainability of manufacturing systems worldwide. Against this backdrop, industrial chain resilience—defined as the capacity to withstand disruptions, achieve rapid recovery, and even undergo structural upgrading—has emerged as a critical metric for assessing a nation’s industrial competitiveness and economic security^1^. As the world’s largest manufacturing nation, enhancing industrial chain resilience is not only an intrinsic imperative for navigating uncertainty and safeguarding industrial security, but also a strategic cornerstone for propelling manufacturing towards higher-value segments of global value chains and achieving high-quality development^2^. However, existing research predominantly focuses on conceptual frameworks or macro-policy discussions regarding resilience, lacking systematic empirical validation grounded in specific technology-driven pathways. Particularly, the quantitative impact of artificial intelligence—a new generation of general-purpose technology—on industrial chain resilience remains under-explored. Therefore, clarifying the intrinsic relationship and operational mechanisms between artificial intelligence and the resilience of manufacturing industrial chains holds significant theoretical value and practical urgency.

Artificial intelligence, as the core driving force of the new round of technological revolution and industrial transformation, is deeply integrating data, algorithms and computing power to comprehensively penetrate all aspects of manufacturing, including research and development design, production manufacturing, operation management and supply chain collaboration^3^. Through intelligent prediction optimization, flexible automation, precise resource allocation and cross-domain knowledge integration, it not only enhances the efficiency of individual links, but also strengthens the overall coordination and adaptive ability of the industrial chain at the system level^4^. This technological empowerment may enhance the resilience, recovery ability and evolution ability of the industrial chain from multiple dimensions such as reinforcing key nodes, optimizing the global network, and stimulating innovation and iteration^5^. Exploring the "technology-system" synergy effect between artificial intelligence and industrial chain resilience is not simply discussing the application of technology itself, but focuses on how artificial intelligence reshapes the underlying logic and interaction patterns of industrial organization. This is crucial for understanding the new source of industrial competitiveness in the digital era and formulating precise industrial technology policies.

Existing research on industrial chain resilience has established a multi-layered theoretical framework: in defining its essence, academia generally adopts a dynamic capability perspective, deconstructing industrial chain resilience into four dimensions: the buffering capacity to withstand shocks, the recovery capacity following disruptions, the adaptive capacity to adjust to environmental changes, and the evolutionary capacity to achieve value chain leapfrogging^6^. This conceptual system not only addresses short-term risk management but also emphasises the evolutionary trajectory of systemic enhancement through structural optimisation and technological diffusion within the long-term development of industrial chains. Regarding influencing factors, research indicates that industrial chain resilience is shaped by multidimensional elements including technological foundations, network structures, institutional environments, and factor allocation^7–9^. Among these, digital and intelligent transformation—as the current key technological drivers—are profoundly reshaping the logic and manifestations of manufacturing resilience by reconfiguring production processes, optimising supply chain coordination, and empowering innovation networks^10^. Regarding measurement methodologies, international research has developed simulation models based on complex systems theory, supply chain vulnerability indices, and multi-indicator evaluation systems, emphasising the identification of critical nodes and transmission pathways through dynamic simulation^11,12^. Domestic research, however, has increasingly integrated the characteristics of China’s industrial system. By constructing multidimensional indicator systems encompassing scale, efficiency, innovation, and sustainability, and employing empirical quantification methods such as principal component analysis and entropy-weighted TOPSIS, it has progressively established an assessment paradigm adapted to local contexts^13^. These studies lay a crucial foundation for deepening our understanding of the theoretical underpinnings and practical manifestations of industrial chain resilience, while also providing a necessary analytical starting point for further exploring how emerging technologies such as artificial intelligence can be embedded within and empower this complex system.

Research on the interplay between artificial intelligence and manufacturing resilience exhibits both complementary and divergent approaches domestically and internationally. Scholars in Europe and America pioneered the ‘intelligent supply chain’ perspective, emphasising how AI significantly enhances supply chain visibility and responsiveness through strengthened predictive analytics, optimised resource allocation, and flexible production^14^. For instance, empirical studies under Germany’s ‘Industry 4.0’ framework demonstrate that AI-driven real-time monitoring and autonomous decision-making systems effectively mitigate operational fluctuations caused by logistics disruptions or sudden demand shifts^15^. Corresponding American research highlights the pivotal role of data fusion and algorithmic models in risk early warning and resilient scheduling, particularly revealing diminishing marginal returns in technology-enabled advancements within high-end manufacturing sectors^16^. Scholars in South Korea and Japan further examine AI’s collaborative control and fault-healing capabilities within complex industrial chains like semiconductors and automobiles, underscoring its value in maintaining stability at critical technological junctures^17^. In contrast, domestic research, while drawing on international theories, places greater emphasis on analysing China’s industrial system characteristics and policy context. Recent literature generally acknowledges that AI not only directly enhances industrial chain resilience by advancing intelligent manufacturing processes and precision supply chain management, but also exerts long-term effects through indirect pathways such as facilitating technological innovation diffusion and optimising regional industrial specialisation^18^. Empirically, domestic scholars have utilised inter-provincial or sectoral panel data to validate AI’s significant positive impact on manufacturing industrial chain resilience, noting that this effect is more pronounced in eastern regions, areas with high digital economy levels, and regions with high urbanization rates^19^. However, compared with international research, domestic studies remain deficient in modelling and testing across areas such as cross-industrial chain dynamic simulation, long-term resilience evolution, and the interactive mechanisms between artificial intelligence and institutional environments. There is a particular lack of systematic reference to the ‘technology-institutional-organisational’ coordination framework adopted in Europe and the United States.

This study, grounded in provincial-level panel data from China, adopts a ‘technology-system’ synergy perspective to construct a multi-level comprehensive measurement framework encompassing artificial intelligence infrastructure, industrial inputs, and outputs. Employing principal component analysis, it systematically measures the resilience of manufacturing industrial chains. This approach breaks away from previous research paradigms often confined to theoretical deduction or qualitative analysis, providing new methodological foundations for quantitative exploration in related fields. In theoretical construction and empirical design, this study not only proposes the research hypothesis that artificial intelligence directly influences industrial chain resilience but also systematically constructs a series of econometric models—including benchmark regression, mediation effects, moderation effects, and threshold effects—based on panel data from 30 Chinese provinces spanning 2012–2023. This comprehensively examines the impact mechanisms, transmission pathways, and nonlinear characteristics of artificial intelligence. Rigorous robustness checks and endogeneity treatments—including subsample analysis, variable measurement substitution, simultaneous equation models, and instrumental variable methods—ensure the reliability of core findings. The study’s innovations are primarily manifested in two aspects: Firstly, in mechanism analysis, it not only verifies the mediating role of economic development but also innovatively introduces urbanization rate as a moderating variable. It further reveals the nonlinear threshold characteristics of AI’s impact on industrial chain resilience, discovering that its promotional effect exhibits a ‘diminishing marginal returns’ pattern as the level of data element development increases, thereby deepening our understanding of the complex dynamic relationship between the two. Secondly, in exploring heterogeneity, regional and quantile regression analyses reveal significant spatial variations and conditional characteristics in AI’s enabling effects. Specifically, effect intensity exhibits a pattern of ‘strongest in the east, next strongest in the west, and least pronounced in the centre,’ with effects intensifying as industrial chain resilience increases, demonstrating a Matthew effect where ‘the strong grow stronger.’ In summary, this study systematically elucidates the intrinsic logic and multifaceted pathways through which artificial intelligence enhances the resilience of manufacturing industrial chains. This is achieved by constructing a comprehensive indicator system, designing a multi-model empirical verification chain, and conducting in-depth analysis across multiple dimensions including mechanisms, moderation, thresholds, and heterogeneity. The research provides an academic reference with both theoretical depth and empirical support for understanding how digital technologies drive industrial system upgrading.

## Theoretical analysis and research hypotheses

### Direct mechanisms of artificial intelligence’s impact on manufacturing supply chain resilience

As a disruptive general-purpose technology, artificial intelligence is systematically reshaping the foundational operational logic of manufacturing through the deep integration of data, algorithms, and computational power. Its direct empowerment of manufacturing supply chain resilience centres on reconstructing information processing paradigms, optimising decision-making processes, and enhancing systemic adaptive capabilities. This comprehensively elevates the supply chain’s capacity for resistance, recovery, adaptation, and evolution when confronting internal and external shocks.

Firstly, artificial intelligence enhances the industrial chain’s capabilities for situational awareness and proactive defence by establishing a comprehensive perception and intelligent early-warning network^20^. Through multimodal data fusion leveraging IoT sensors, supply chain collaboration platforms, and external data sources, machine learning algorithms enable real-time monitoring and pattern recognition of critical supply chain node statuses, logistics fluctuations, market demand anomalies, and potential risks. This intelligent alert mechanism, transcending traditional threshold-based approaches, enables the system to initiate contingency plans and pre-allocate resources before or during the initial stages of an impact. This transforms passive response into proactive defence, significantly enhancing the supply chain’s buffering capacity. Secondly, AI-driven dynamic optimisation and autonomous decision-making substantially boost the supply chain’s ‘agile response’ and ‘rapid recovery’ capabilities^5^. When confronting supply disruptions or sudden demand shifts, intelligent scheduling systems based on reinforcement learning and operational optimisation rapidly generate and iterate recovery plans under multi-objective constraints such as cost, time, and resources. This enables dynamic reorganisation of production schedules, inventory allocation, and logistics routes. Not only does this compress the post-impact response window, but it also reduces friction costs during recovery by enhancing resource allocation precision, thereby ensuring rapid restoration of industrial chain continuity. Finally, AI-enabled intelligent production processes and flexible reconfiguration lay the foundation for the industrial chain’s ‘dynamic adaptation’ and ‘structural evolution’^21^. Technologies such as computer vision, industrial robotics, and digital twins drive the transformation of production lines into modular, reconfigurable flexible manufacturing cells. This enables manufacturing systems to rapidly adjust process flows and equipment collaboration models in response to product order changes or capacity constraints. This inherent flexibility enables industrial chains not only to adapt to short-term, known fluctuations but also to provide organisational and process-level evolvability for addressing long-term, structural transformations.

Based on the aforementioned theoretical analysis, this paper proposes the following research hypothesis:

H1: The development of artificial intelligence exerts a significant direct positive effect on the resilience of manufacturing industrial chains.

### Indirect Mechanisms of artificial intelligence’s impact on manufacturing supply chain resilience

The enhancement of supply chain resilience through artificial intelligence manifests not only as direct technological empowerment but also exerts profound indirect effects by elevating regional economic development levels. This transmission pathway is grounded in new growth theory and regional innovation system theory. As a general-purpose technology, the penetration and application of artificial intelligence can significantly drive regional economic growth by enhancing total factor productivity, optimising resource allocation efficiency, and catalysing new business models and formats^22^. The resulting elevation in economic development levels thereby establishes a more robust foundation and broader scope for manufacturing industrial chain resilience, constituting a critical indirect transmission channel.

Economic development provides multidimensional support for enhancing industrial chain resilience: Firstly, higher economic development levels typically imply stronger fiscal capacity and more comprehensive public infrastructure^23^. Local governments can allocate more resources to build and maintain high-standard infrastructure such as transportation, logistics, and information communication facilities, and establish more effective industrial public service platforms and emergency management systems. This directly reduces the systemic basic risks in the operation of the industrial chain and enhances its collective action ability to cope with shocks. Secondly, economic growth is accompanied by industrial structure optimization and market capacity expansion^24^. A more diversified and advanced industrial structure can reduce the region’s reliance on a single manufacturing chain and form a risk-distributing “buffer zone”; at the same time, the vast local market provides a stable demand base for manufacturing enterprises, enhancing their demand-side stability when facing external demand fluctuations. Thirdly, economic development is often positively correlated with human capital accumulation, financial deepening, and the activity of technological innovation^25^. Abundant high-quality labor force, convenient financing channels, and an active innovation ecosystem enable manufacturing enterprises to have the ability for technological transformation, digital upgrading, and supply chain collaborative innovation, thereby continuously enhancing their adaptability and evolutionary ability to cope with long-term structural challenges.

Therefore, artificial intelligence indirectly fosters a more supportive macroeconomic environment and economic ecosystem for the resilience of manufacturing industrial chains by driving economic growth. This indirect effect is not a simple linear transmission but rather a chain reaction process whereby artificial intelligence: → promotes regional economic growth through technological diffusion and productivity enhancement; → improves industrial foundational conditions, optimises industrial structures, and stimulates innovation vitality; → ultimately enhances the systemic resilience of manufacturing industrial chains. Based on the aforementioned theoretical analysis, this paper proposes the following research hypothesis:

H2: Artificial intelligence exerts a positive, indirect influence on the resilience of manufacturing industrial chains by promoting regional economic development levels. That is, economic development levels mediate the impact of artificial intelligence on the resilience of manufacturing industrial chains.

### Analysis of the modulating effects of urbanization rates

Urbanization signifies not merely the spatial concentration of population and economic activity, but also represents the deep integration of advanced factors such as capital, information, technology, and talent, alongside the continuous evolution of innovation ecosystems. This constitutes a crucial contextual variable in enhancing the resilience of industrial chains through artificial intelligence technology. The urbanization rate may exert a positive moderating influence on AI’s impact upon the resilience of manufacturing industrial chains, with its operational logic manifesting across three primary dimensions:

Firstly, the deepening of factor markets and enhancement of infrastructure accompanying urbanization amplify both the breadth and depth of AI technology application^26^. Regions with a high urbanization rate usually have more developed digital infrastructure, denser talent reserves, and more active venture capital. These conditions provide fertile soil for the research, dissemination and commercialization of artificial intelligence technologies. The improvement of infrastructure reduces the marginal cost for enterprises to adopt artificial intelligence technologies, while the concentration of human capital enhances the absorption, digestion and re-innovation capabilities of the technology. Together, these ensure that the enabling effect of artificial intelligence can be fully exerted, thereby more effectively transforming into the risk warning, agile response and structural reorganization capabilities of the industrial chain. Secondly, the strengthened industrial linkage promoted by urbanization and the knowledge spillover effect optimize the transmission path of artificial intelligence within the industrial chain. Urbanization promotes the geographical proximity and functional coupling of manufacturing and production-related services, forming a more collaborative local production network^27^. In this networked ecosystem, artificial intelligence technologies are more likely to expand from individual enterprise applications to the coordinated upstream and downstream of the industrial chain, for example, intelligent algorithms can perform global optimization based on a more complete supply chain data pool, and intelligent logistics systems can more efficiently connect enterprises within the park. This strengthening of industrial linkage based on spatial agglomeration enhances the collaborative application of technology and the cross-organizational spillover of knowledge, enabling the enhancement of resilience through artificial intelligence to rise from the micro level of enterprises to the meso-level system of the industrial chain. Finally, the innovation of governance models and the optimization of the institutional environment caused by urbanization provide necessary institutional guarantees and collaborative governance frameworks for the empowerment of resilience by artificial intelligence. The urbanization process often accompanies the modernization transformation of local government governance capabilities, including more efficient government services, more transparent market rules, and more proactive policy support for digital transformation. This creates a more favorable institutional environment for the standardized application of artificial intelligence technologies, data security and privacy protection, as well as the formulation and implementation of relevant technical regulations. A good institutional environment can reduce the uncertainty of technology application, stimulate enterprises to make long-term and strategic intelligent investments, and thereby ensure the stability and sustainability of the promoting effect of artificial intelligence on the resilience of the industrial chain.

Based on the above analysis, urbanization can effectively amplify the positive impact of artificial intelligence on the resilience of manufacturing industrial chains by improving the foundational conditions for technological application, strengthening the network effects of industrial synergy, and optimising the governance environment through institutional support. Therefore, this paper proposes the following moderating effect hypothesis:

H3: urbanization rates exert a positive moderating effect on the process whereby artificial intelligence enhances the resilience of manufacturing industrial chains. That is, the higher the level of urbanization, the stronger the enhancing effect of artificial intelligence on the resilience of manufacturing industrial chains.

### Threshold effects of data element development levels

As a strategic technology spearheading the new wave of technological revolution and industrial transformation, artificial intelligence’s role in enhancing the resilience of manufacturing supply chains hinges upon the support of foundational elements and enabling environments. Data elements, as the core productive factors of the digital era, directly influence the training efficacy, model accuracy, and application depth of AI algorithms through their scale, quality, circulation efficiency, and governance standards^28^. From a theoretical perspective, data serves not only as the ‘fuel’ for iterative AI optimisation but also as the foundation for constructing industrial digital ecosystems and enabling systematic intelligent decision-making. Consequently, the enabling effect of AI on industrial chain resilience is not linear or singular; it is likely modulated by the development level of data elements, exhibiting pronounced threshold characteristics.

During the early stages of data development, regional data collection infrastructure remains underdeveloped, data standardisation is low, and mechanisms for circulation and sharing are absent. At this stage, AI applications are often constrained by insufficient data volume, inconsistent quality, and fragmented use cases. While AI technology can still deliver efficiency gains through localised optimisation and process automation, its potential for enabling collaborative early warning systems, elastic scheduling, and dynamic adaptation across industrial chains is suppressed, resulting in relatively limited contributions to resilience. However, when data element development surpasses a critical threshold – signifying the establishment of fundamental data infrastructure, the initial maturation of data governance systems, and the effective operation of cross-entity data sharing mechanisms – high-quality, highly fluid, and highly credible data flows can fully drive AI models to perform precise predictions, intelligent matching, and adaptive optimisation. Artificial intelligence not only enhances efficiency at individual stages but also enables data-driven real-time perception, dynamic assessment, and collaborative response across the entire industrial chain. This significantly strengthens the system’s overall risk resistance and recovery resilience.

Based on the foregoing analysis, this paper proposes the following research hypothesis:

H4: The development level of data elements exerts a positive threshold effect on the enabling impact of artificial intelligence. Specifically, as the development level of data elements increases, the promotional effect of artificial intelligence on the resilience of the manufacturing industrial chain exhibits a non-linear characteristic of increasing marginal returns.

**Fig. 1:**
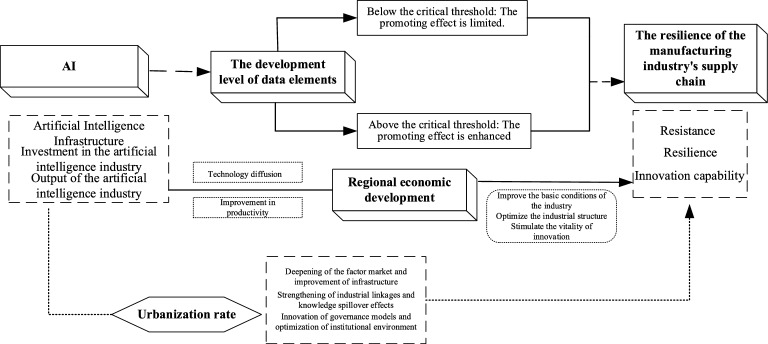
Mechanism analysis diagram.

To explain this mechanism more clearly and intuitively, the mechanism diagram is shown as Fig. 1 below.

## Research design

### Model construction

#### Benchmark model construction

To empirically examine the impact of artificial intelligence (AI) development on the resilience of manufacturing industrial chains, this study establishes the following baseline regression model:

1 $$Resi_{it} = \alpha_{0} + \alpha_{1} AI_{it} + \beta Cons_{it} + \mu_{i} + \delta_{t} + \varepsilon_{it}$$

wherein, *i* denotes region, *t* denotes year, *Resi* represents the dependent variable “resilience of the manufacturing industry chain”, and *AI* denotes the core explanatory variable “level of artificial intelligence”. ${\alpha }_{0},{ \alpha }_{1}, \beta$ denote the intercept term, the estimated coefficient of the core explanatory variable on the dependent variable, and the estimated coefficient of the control variable on the dependent variable respectively.

#### Mediating effect model

To examine whether artificial intelligence enhances the resilience of manufacturing industrial chains by promoting regional economic development (*REDL*), the stepwise regression method for mediating effect is constructed:

2 $${REDL}_{it}={\alpha }_{0}+{\alpha }_{1}{AI}_{it}+\beta {Cons}_{it}+{\mu }_{i}+{\delta }_{t}+{\varepsilon }_{it}$$

#### Moderating effect model

To examine the moderating effect of urbanization rate (*URB_per*), the following model is constructed:

3 $${Resi}_{it}={\alpha }_{0}+{\alpha }_{1}{AI}_{it}+{\alpha }_{2}{URB\_per}_{it}+{\alpha }_{3}{AI}_{it}\cdot {URB\_per}_{it}+\beta {Cons}_{it}+{\mu }_{i}+{\delta }_{t}+{\varepsilon }_{it}$$

wherein, *URB_per* denotes the moderator variable, while *AI·URB_per* represents the interaction term measuring the moderating effect.

#### Threshold effect model

To examine the non-linear relationship between artificial intelligence and the resilience of the manufacturing industry chain, this paper selects the level of development of data elements as the threshold variable T and constructs a threshold model. The model is formulated as follows:

$${Resi}_{it}={\alpha }_{0}+{\alpha }_{1}{AI}_{it}\cdot I\left({T}_{it}<{\eta }_{1}\right)+{\alpha }_{2}{AI}_{it}\cdot I\left({\eta }_{1}\le {T}_{it}<{\eta }_{2}\right)+$$

4 $${\alpha }_{3}{AI}_{it}\cdot I\left({T}_{it}\ge {\eta }_{2}\right)+\beta {Cons}_{it}+{\mu }_{i}+{\delta }_{t}+{\varepsilon }_{it}$$

wherein, *T* denotes the threshold variable, and *η* represents the threshold value to be estimated. When the condition within the parentheses is satisfied, the function *I(·)* is assigned the value 1; otherwise, it is assigned the value 0.

### Variable selection

#### Dependent variable

The article, drawing upon the concept of evolutionary resilience, defines industrial chain resilience as the capacity of an industrial chain to maintain stability, address deficiencies, and enhance strength when confronted with chronic pressures or acute shocks^29^. This resilience manifests primarily through three dimensions: resistance, recovery, and innovation. This resilience manifests primarily through three dimensions: resistance, recovery, and innovation. Manufacturing industrial chain resilience constitutes a vital subset of industrial chain resilience, with its three dimensions manifested as follows: The resilience dimension primarily reflects the manufacturing industrial chain’s stability and capacity to prevent disruption when confronting external pressures or shocks, i.e., its ‘chain stabilisation’ capability. The strength of resilience is often closely linked to the initial development trajectory of the industrial chain, particularly the rationality of its scale and structure. Within manufacturing supply chains, the resilience dimension manifests as the chain’s adaptability following pressures or shocks, alongside its capacity for rapid recovery to its original state – commonly termed ‘supply chain restoration capability’. Resilience is determined by both the speed and extent of a chain’s recovery to stability post-impact, while its developmental efficiency and flexible adjustment capabilities also play crucial roles. The dimension of transformative capacity reveals a manufacturing industrial chain’s ability to achieve transformation and upgrading by renewing its operational models, particularly its capacity to pioneer new development pathways. The ability to achieve ‘pathway breakthroughs’ is a key factor in enhancing a manufacturing industrial chain’s transformative capacity. The transformative potential of the manufacturing industrial chain, coupled with the support of the external environment, jointly determine the magnitude of its transformative capacity. Thus, these three dimensions—resistance, resilience, and transformation capacity—correspond respectively to an industrial chain’s stability, recovery capability, and transformation capability in the face of shocks, constituting the core elements of industrial chain resilience. In summary, this paper integrates these three capabilities by constructing an indicator system as shown in (Table 1) and employing principal component analysis to measure the explained variable: manufacturing industrial chain resilience (*Resi*).

**Table 1:** Manufacturing industrial chain resilience level indicators.

Primary indicator	Secondary indicator	Tertiary indicator	Calculation method
Resistance	Prevention capacity	Concentration Ratio	Herfindahl–Hirschman index (HHI)
		Foreign capital risk degree	Total foreign assets of above-scale industrial enterprises
			Total realized assets of above-scale industrial enterprises
			Total assets of industrial enterprises with foreign investment and Hong Kong, Macao, and Taiwan investment
		Enterprise nationalization	Total assets of above-scale state-controlled industrial enterprises
			Total assets of above-scale industrial enterprises
			Total assets of state-controlled industrial enterprises (industrial enterprise total assets)
		Main business profit margin	Total profit of the manufacturing industry
			Main business income of the manufacturing industry
			Operating profit of the manufacturing industry
	Bearing capacity	Enterprise loss rate	Total loss of the manufacturing industry
			Operating profit of the manufacturing industry / Main business income of the manufacturing industry
			Total loss of the manufacturing industry / Total profit of the manufacturing industry
		Enterprise scale	Total assets of the manufacturing industry
Resilience	Adaptability	Industrial structure foundation	Proportion of main business income of high-tech manufacturing in the manufacturing industry
		Industrial synergy	EG Index of co-agglomeration between manufacturing and producer services
		Financial synergy	Ratio of total loans of banking institutions to GDP
		Number of top 500 enterprises	Number of Chinese manufacturing enterprises in the Top 500
	Restoration capacity	Human capital investment	Average employment in the manufacturing industry
			Number of legal entities in the manufacturing industry
		Labor productivity	Industrial added value
			Industrial added value / Average employment in the manufacturing industry
		New Product Profit Contribution	Proportion of new product sales income of above-scale industrial enterprises in main business income
Innovation power	Transformation capacity	Green Transformation	Energy consumption per unit of regional GDP
			Electricity consumption per unit of industrial added value
	Innovation capacity	Innovation input	Full-time equivalent of R&D personnel in above-scale industrial enterprises
			Internal R&D expenditure of above-scale industrial enterprises
		Innovation output	Effective industrial inventions
			Internal R&D expenditure of industrial enterprises / Main business income of the manufacturing industry

#### Independent variable

The advancement of artificial intelligence, particularly driven by large-scale pre-trained models, heterogeneous computing clusters, and the engineering application of cutting-edge algorithms, is systematically reshaping the structure and operational mechanisms of the manufacturing industry chain. It functions not merely as a technical tool embedded within production processes, but as a novel production paradigm characterised by ‘data-driven, intelligent decision-making, and autonomous collaboration,’ profoundly influencing every stage of the industrial chain’s lifecycle. At the manufacturing end, machine vision-based intelligent quality inspection and predictive maintenance achieve closed-loop optimisation of quality control. Within the supply chain, demand-sensing and risk-warning models leveraging natural language processing and knowledge graphs significantly enhance the system’s proactive responsiveness. On the consumption and service side, personalised recommendation systems and intelligent customer service platforms facilitate precise matching between supply and demand. These pervasive intelligent applications collectively form the key enabling pathway for enhancing industrial chain resilience—namely, by strengthening real-time perception, elastic scheduling, and dynamic optimisation capabilities to elevate the chain’s resistance, recovery capacity, and adaptability in the face of uncertainty.

To scientifically measure this multidimensional, systematic technological empowerment, this paper constructs a comprehensive evaluation framework following the logical chain of ‘foundational support—process investment—outcome transformation’. Concrete indicators are selected across three dimensions: artificial intelligence infrastructure^30^, artificial intelligence industry investment^31^, and artificial intelligence industry output^32^. This aims to comprehensively depict the developmental realities of artificial intelligence across regions, from technological reserves to industrial integration. Building upon this foundation, principal component analysis is employed to reduce the dimensionality of the multi-dimensional indicators, synthesising a composite score for AI development levels. This provides a scientifically sound and operationally viable metric for subsequent quantitative analysis of the causal relationship between AI development and industrial chain resilience. The indicator system is shown in (Table 2).

**Table 2:** Artificial intelligence development level indicators.

Primary indicator	Secondary indicator	Calculation method
Artificial intelligence infrastructure	Internet broadband penetration rate	Number of internet broadband access ports / Regional permanent population
	Information technology infrastructure	Long-distance optical cable length / National territorial area
	Mobile communication infrastructure	Number of mobile phones per 100 people
	Artificial intelligence attention	Weighted sum of search frequencies of AI keywords on Baidu
	Artificial intelligence enterprise proportion	Number of AI-related industry enterprises / Number of regional enterprise legal entities
Artificial intelligence industry input	Artificial intelligence talent input	Number of employees in AI-related industries / Number of regional employees
	Artificial intelligence fixed asset investment proportion	Fixed asset investment in AI-related industries / Regional fixed asset investment
	Artificial intelligence R&D expenditure proportion	R&D expenditure of AI-related industries / Regional R&D expenditure
Artificial intelligence industry output	Artificial intelligence Main business income proportion	Main business income of AI-related industries / (Main business income of above-scale industrial enterprises + Main business income of above-scale industrial enterprises)
	Artificial intelligence patent output proportion	Total AI patents retrieved from the State Intellectual Property Office / Regional patent applications

#### Mediating variable

Regional economic development level (*REDL*): To examine the mediating pathway through which artificial intelligence enhances industrial chain resilience by promoting regional macroeconomic growth, this study employs per capita regional GDP as a proxy variable for regional economic development level.

#### Moderating variable

Urbanization rate (*URB_per*): To explore the moderating role of regional urbanization in AI’s influence on industrial chain resilience, this study employs the proportion of urban population relative to total population as the measure of urbanization rate.

#### Threshold variable

Data element development level (*DEDL*): An integrated evaluation framework is constructed across four dimensions—data application environment, dissemination and sharing, development and application, and element management. Entropy-based methods systematically calculate each province’s data element level, enabling a comprehensive and structured reflection of regional data empowerment capabilities at different developmental stages. Specific indicator construction is detailed in (Table 3).

**Table 3:** Indicator system for the development level of data elements.

Primary indicator	Secondary indicator	Tertiary indicator	Specific definition of indicator
Data element development level	Data dissemination and sharing	Number of domain names	Direct data
		Number of web pages	Direct data
		Total telecommunications business volume	Direct data
		Total postal business volume	Direct data
		Mobile phone switching capacity	Direct data
		Mobile phone penetration rate	Direct data
		Mobile internet users	Direct data
		IPv4 address count	Direct data
	Data application environment	Optical cable line length	Direct data
		Postal business outlets	Direct data
		Internet broadband access ports	Direct data
		Broadcast program popularization degree	Comprehensive population coverage rate of broadcast programs
		TV program popularization degree	Comprehensive population coverage rate of TV programs
		Fiscal science, technology and education expenditure	Local fiscal science & technology expenditure + local fiscal education expenditure
		Enterprise information infrastructure level	Number of websites per 100 enterprises
		Number of computers per 100 people	Direct data
		Higher education resources	Number of regular higher education institutions
		Digital technology market scale	Technology market turnover
		Employees in information transmission, software and information technology services (urban units)	Direct data
	Data element management	High-tech industry R&D personnel	Full-time equivalent of high-tech industry R&D personnel
		High-tech industry R&D expenditure	Internal R&D expenditure of high-tech industry
		High-tech industry patent applications	Number of high-tech industry patent applications
		High-tech industry institutions	Number of R&D institutions run by high-tech enterprises
	Data development and application	Enterprise informatization	Proportion of enterprises with E-commerce transaction activities
		Digital inclusive finance	Digital inclusive finance index
		Express business development	Express business income
		E-commerce scale	E-commerce sales volume
		Product quality qualification rate	Direct data
		Residents’ transportation and communication expenditure ratio	Per capita household expenditure on transportation and communication/per capita household consumption expenditure
		Number of legal entities in information transmission, software and information technology services	Direct data

#### Control variables

To more accurately identify the impact of artificial intelligence on the resilience of the manufacturing industry chain, this paper incorporates the following control variables into the model to mitigate the influence of other regional characteristics:

1. Urban resident income level (URB_inc): Measured by per capita disposable income of urban residents to control for regional consumption capacity and market demand;
2. Research and development (R&D) Intensity: Measured by the natural logarithm of internal R&D expenditure as a proportion of regional GDP, to control for the influence of regional technological innovation activities;
3. Level of service sector development (ind_third): Measured by the proportion of tertiary industry value added in regional GDP, to control for the impact of regional industrial structure upgrading;
4. Macro tax burden level (TAX): Measured by the ratio of tax revenue to regional GDP, to control for the impact of regional fiscal policies and business environments.

### Data sources

This study utilizes panel data from 30 Chinese provinces (excluding Tibet, Hong Kong, Macao, and Taiwan) covering the period 2012–2023. The primary data sources include: the National Bureau of Statistics of China, the Development Research Center of the State Council (DRC), the EPS Data Platform, the CSMAR database, the China Information Industry Yearbook, the China Industrial Statistics Yearbook, the China Science and Technology Statistics Yearbook, and the High-Tech Industry Statistics Yearbook. Minor missing values were imputed using linear interpolation. Variable abbreviations are shown in (Table 4).

**Table 4:** List of abbreviations.

Abbreviation	Full name
*Resi*	manufacturing industrial chain resilience
*AI*	Artificial intelligence development
*REDL*	Regional economic development level
*URB_per*	Urbanization rate
*DEDL*	Data element development level
*URB_inc*	Urban resident income level
*R&D*	Research and development
*ind_third*	Level of service sector development
*TAX*	Macro tax burden level

## Empirical findings and analysis

### Benchmark regression analysis

The primary objective of this study is to examine the direct impact of artificial intelligence (AI) on manufacturing supply chain resilience. To mitigate potential multicollinearity and isolate the independent effect of AI, we employ a two-way fixed effects model with stepwise regression. The results, presented in Table 5, demonstrate the robustness of AI’s impact as control variables are sequentially introduced.

**Table 5:** Benchmark regression results.

	(1)	(2)	(3)	(4)	(5)
	*Resi*	*Resi*	*Resi*	*Resi*	*Resi*
*AI*	2.951***	2.536***	1.174***	1.173***	1.294***
	(12.55)	(10.55)	(5.90)	(5.89)	(6.26)
*URB_inc*		0.000***	0.000***	0.000***	0.000***
		(5.12)	(8.15)	(8.14)	(8.22)
*R&D*			0.002***	0.002***	0.002***
			(15.88)	(15.06)	(14.45)
*TAX*				0.813	0.126
				(0.21)	(0.03)
*Ind_third*					0.030**
					(2.07)
*_cons*	-5.532***	-6.675***	-10.803***	-10.852***	-12.095***
	(-15.32)	(-16.16)	(-26.78)	(-23.24)	(-15.95)
N	360	360	360	360	360
R^2^	0.692	0.715	0.842	0.842	0.844

*,** and *** denote significance at the 10, 5 and 1% levels respectively; values in parentheses represent robust standard errors; the same applies below.

#### Independent promoting effect of artificial intelligence

In Column (1), where only artificial intelligence (AI) is included as an explanatory variable, its estimated coefficient is 2.951, significantly positive at the 1% level. This indicates that, without controlling for other factors, the development of artificial intelligence exerts a significant positive influence on the resilience of the manufacturing industrial chain, preliminarily supporting Hypothesis H1: that artificial intelligence can enhance industrial chain resilience.

#### Robustness test following stepwise inclusion of control variables

To further control for interference from regional socio-economic characteristics, Models (2) to (5) progressively incorporated urban residents’ income level (*URB_inc*), R&D intensity (*R&D*), macro tax burden level (*TAX*), and service sector development level (*Ind_third*). Results show that the coefficient for artificial intelligence consistently remained positive and significant at least at the 1% level, fluctuating between 1.173 and 2.951. This outcome indicates that the promotional effect of artificial intelligence on industrial chain resilience did not undergo qualitative change upon introducing other explanatory variables, demonstrating robust stability. Among the control variables, both urban residents’ income level (*URB_inc*) and R&D intensity (*R&D*) were significantly positive at the 1% level, suggesting that enhanced regional consumption capacity and increased innovation investment contribute to building industrial chain resilience. The inclusion of the level of service sector development (*Ind_third*) also yielded a positive and significant effect, suggesting that the evolution of industrial structure towards services may enhance resilience by improving systemic coordination efficiency.

#### Gradual enhancement of model explanatory power

With the progressive introduction of control variables, the model’s coefficient of determination R^2^ increased steadily from 0.692 to 0.844, indicating a continuous strengthening of the model’s explanatory power regarding industrial chain resilience. Notably, the inclusion of R&D intensity and the share of the service sector led to a significant increase in R^2^, reflecting that innovation activities and industrial structure are crucial dimensions influencing resilience. Nevertheless, artificial intelligence consistently remains one of the variables with the strongest explanatory power in the model, highlighting its pivotal role in enhancing industrial chain resilience.

### Robustness tests

To validate the reliability of the benchmark regression results, this paper conducts robustness tests across three dimensions: sample selection, variable control, and measurement methodology.

#### Sample period adjustment test

Considering that the impact of artificial intelligence on the resilience of the manufacturing industry chain may exhibit a certain time lag, and that macroeconomic environments and industrial policies vary significantly across different periods, sensitivity testing is conducted on the sample period to eliminate interference from structural factors specific to particular timeframes. Given that the period from 2015 to 2020 witnessed the successive introduction of major national strategies such as Made in China 2025 and the New Generation Artificial Intelligence Development Plan, marking a critical phase for the large-scale application and deep industrial integration of AI technologies, while also encompassing pivotal junctures in domestic and international economic conditions and supply chain configurations, this study re-conducted regression analysis using a subsample from 2015 to 2020. As shown in Table 6 (1), the estimated coefficient for the core explanatory variable ‘artificial intelligence’ remains significantly positive at the 1% level. This indicates that, even after controlling for policy and economic structural heterogeneity across different periods, the positive effect of artificial intelligence on the resilience of the manufacturing industry chain remains robust.

**Table 6:** Robustness tests.

	(1)	(2)	(3)
	*Resi*	*Resi*	*Resi*
*AI*	1.611***	1.207***	0.173***
	(5.43)	(10.06)	(10.76)
*URB_inc*	0.000***	-0.000***	0.000**
	(2.69)	(-6.73)	(2.14)
*R&D*	0.002***	-0.001***	0.002***
	(7.70)	(-2.81)	(8.61)
*TAX*	-0.165	1.019	-0.734
	(-0.03)	(0.41)	(-0.21)
*Ind_third*	-0.003	-0.014	-0.006
	(-0.14)	(-1.63)	(-0.49)
*retail*		0.000***	
		(5.39)	
*loan*		0.000***	
		(20.32)	
*land*		0.000	
		(0.00)	
*GOV*		-0.000	
		(-1.14)	
*_cons*	-9.546***	-1.170*	-6.251***
	(-7.31)	(-1.72)	(-7.96)
N	180	360	360
R^2^	0.734	0.948	0.872

#### Omitted variable control test

Although the benchmark regression controlled for multiple regional characteristic variables, the possibility of omitting important explanatory variables remains. To further mitigate endogeneity bias caused by omitted variables, this paper introduces the following additional provincial-level control variables to the benchmark model, drawing on relevant research: Total retail sales of consumer goods (*retail*), to control for regional domestic demand and market vitality; total outstanding loans in both domestic and foreign currencies from financial institutions (*loan*), reflecting regional financial deepening and credit supply levels; administrative land area (*land*), controlling for regional resource endowments and scale differences; and government fiscal expenditure as a percentage of GDP (*GOV*), depicting the degree of government intervention and the macro-policy environment. The regression results are presented in Table 6 (Column 2). After incorporating these variables, the coefficient for artificial intelligence remains significantly positive and stable in value. This indicates that the core conclusions are insensitive to the addition or removal of control variables, further reducing estimation bias caused by omitted variables.

#### Test of core variable measurement method replacement

To eliminate interference from the specificity of the dependent variable measurement method, this study replaces the measurement method for the core explanatory variable, the level of artificial intelligence development. In the benchmark regression, principal component analysis was employed to construct the composite indicator. For robustness testing, the entropy method was used to re-measure AI development levels. The entropy method is a comprehensive evaluation technique based on objectively weighting indicators according to their degree of dispersion, thereby mitigating biases associated with subjective weighting. As shown in column (3) of Table 6, the estimated coefficient for the AI variable measured using the entropy method remains significantly positive at the 1% level. This indicates that regardless of whether principal component analysis or the entropy method is employed, the positive promotional effect of AI on the resilience of the manufacturing industry chain remains robust, with the conclusion unaffected by specific measurement methods.

### Endogeneity testing and treatment

To ensure the robustness and reliability of our findings, this study systematically addresses potential endogeneity concerns. Specifically, we focus on three primary sources: simultaneity (reverse causality), omitted variable bias, and measurement error. The following three methods are employed to identify and mitigate these issues:

#### Simultaneous equation model estimation

To examine potential bidirectional causality between artificial intelligence (AI) and the resilience of the manufacturing industry chain (*Resi*), this paper constructs a simultaneous equation model comprising two equations. Model (1) serves as the first equation of the simultaneous equation model, while the second equation is defined as ${AI}_{it}={\alpha }_{0}+{\alpha }_{1}{Resi}_{it}+\beta {Cons}_{it}$. In the empirical analysis, the ratio of tertiary to secondary industry value added—which characterises the level of industrial structure upgrading across provinces—is employed as a control variable. Column (1) of Table 7 reports the estimation results of the simultaneous equation model. The coefficient for artificial intelligence remains significantly positive, indicating that the positive promotional effect of artificial intelligence on industrial chain resilience persists even when controlling for the possibility of reverse causality. The benchmark regression results demonstrate robust stability.

**Table 7:** Discussion on endogeneity.

	(1)	(2)	(3)
	*Resi*	*Resi*	*Resi*
*AI*	2.301***	0.042	14.755***
	(9.84)	(1.34)	(4.56)
*URB_inc*	0.000***	0.000***	-0.000**
	(6.08)	(10.17)	(-2.14)
*R&D*	0.002***	0.003***	-0.002*
	(25.40)	(17.92)	(-1.68)
*TAX*	48.486***	1.489	-4.932
	(13.73)	(0.36)	(-0.33)
*Ind_third*	-0.110***	0.005	0.316***
	(-6.73)	(0.31)	(3.67)
*_cons*	-10.483***	-10.798***	-60.445***
	(-20.73)	(-13.99)	(-5.04)
N	360	360	330
R^2^	0.908	0.825	0.846

#### Counterfactual test – constructing a pseudo-treatment variable via random matching

To mitigate endogeneity bias arising from omitted variables or unobservable factors, this study adopts a counterfactual analysis approach by employing random matching to construct a ‘pseudo-AI application’ variable for placebo testing. Specifically, the observed values of the AI variable are randomly reassigned within the sample to generate a pseudo-variable unrelated to actual AI development, which is then substituted into the baseline model for re-estimation. As shown in Column (2) of Table 7, the estimated coefficient of the pseudo variable approaches zero and is statistically insignificant. This indicates that AI’s impact on industrial chain resilience is not driven by random factors or unobservable confounding variables, thereby further supporting the conclusion that AI possesses a genuine causal effect.

#### Instrumental variables method

To further enhance the reliability of causal inference, this study employs the lagged manufacturing industrial chain resilience as an instrumental variable for artificial intelligence. This variable satisfies the correlation requirement—prior resilience may influence current AI development levels through factors such as regional industrial policies and technological accumulation pathways—while also meeting the exogeneity requirement, as prior resilience does not directly affect current resilience levels. The results of the second-stage regression are presented in Column (3) of (Table 7). The coefficient for artificial intelligence remains significantly positive. Furthermore, the Cragg-Donald Wald F statistic of 17.056 exceeds the commonly used critical value of 10, indicating no weak identification issues with the instrumental variable and confirming its validity. This result further validates the robustness and causality of artificial intelligence’s promotional effect on the resilience of the manufacturing industrial chain.

### Testing the mechanism pathway

To delve deeper into the intrinsic transmission mechanism through which artificial intelligence enhances the resilience of manufacturing industry chains, this study focuses on the pivotal mediating role of regional economic development. To validate this mediating pathway, empirical analysis is conducted based on the mediation effect testing model (Eq. 2), with regression results summarised in (Table 8). As shown in Table 8 (1), controlling for a series of relevant variables, the coefficient for artificial intelligence (*AI*) on regional economic development level (*REDL*) is 0.067, which is positively significant at the 10% statistical level. This result confirms that AI exerts a significant promotional effect on regional economic development.

**Table 8:** Mechanism analysis.

	(1)
	*REDL*
*AI*	0.067*
	(1.69)
*URB_inc*	0.000***
	(32.81)
*R&D*	-0.000***
	(-2.69)
*TAX*	0.010
	(0.01)
*Ind_third*	-0.015***
	(-5.45)
*_cons*	-1.559***
	(-10.69)
N	360
R^2^	0.969

Combining this with the direct positive effect of AI on industrial chain resilience (Resi) observed in the baseline regression earlier, alongside the positive impact of AI on regional economic development (*REDL*) here, preliminary evidence for the mediating pathway emerges. This indicates that AI not only directly empowers industrial chain resilience but also indirectly promotes it by stimulating regional economic growth. In other words, the advancement of artificial intelligence enhances the overall economic strength of a region, improves resource allocation capabilities, and invigorates market dynamics. This provides a more robust macroeconomic environment for industrial chains to withstand external shocks, achieve rapid recovery, and undertake structural adjustments. Consequently, it indirectly consolidates and strengthens the resilience of industrial chains themselves.

### Moderation effect test

To verify the moderating role of urbanization rate in the process whereby artificial intelligence influences the resilience of manufacturing industrial chains, this study constructed a moderation effect model incorporating an interaction term. Results indicate that the coefficient for the interaction term between artificial intelligence and urbanization rate (*URB_per·AI*) is 7.194, significantly positive at the 1% level. This demonstrates that urbanization rate significantly enhances the promotional effect of artificial intelligence on industrial chain resilience, supporting research hypothesis H3. Concurrently, the model incorporating the interaction term achieved an R^2^ value of 0.874, demonstrating markedly enhanced explanatory power compared to the baseline model (R^2^ = 0.845). This underscores the significant explanatory value of urbanization’s moderating influence. This finding suggests that in advancing AI-enabled resilience enhancement within manufacturing, considerable emphasis should be placed on the coordinated development of regional urbanization levels. By optimising infrastructure, factor allocation, and institutional environments, the technological potential of AI can be maximised.

### Threshold effect analysis

This paper delves into the non-linear impact mechanism of artificial intelligence on the resilience of manufacturing industrial chains. By introducing the development level of data elements as a threshold variable, it reveals the phased characteristics of their relationship, primarily grounded in the foundational status of data elements as a novel factor of production in the digital era. The efficacy of artificial intelligence technology is highly contingent upon the supply and circulation of high-quality, large-scale data. The development of data elements constitutes a progressive process, evolving from infrastructure deployment to deep governance and application. Consequently, the level of data element development not only reflects the material foundation for artificial intelligence operations but also signifies the maturity of a region’s digital ecosystem Table 9. It is thus well-suited as a threshold variable to characterise the structural transformation in the mechanism of artificial intelligence’s influence.

**Table 9:** Moderation effects.

	(1)	(2)
	*Resi*	*Resi*
*AI*	1.302***	0.570***
	(6.31)	(2.77)
URB_per	-3.887	14.077***
	(-1.37)	(4.23)
URB_per*·AI*		7.194***
		(8.47)
*URB_inc*	0.000***	0.000
	(4.78)	(1.64)
*R&D*	0.003***	0.002***
	(13.33)	(11.84)
*TAX*	-0.045	5.980*
	(-0.01)	(1.66)
*Ind_third*	0.024	0.030**
	(1.60)	(2.20)
*_cons*	-9.710***	-17.463***
	(-5.12)	(-8.99)
N	360	360
R^2^	0.845	0.874

The threshold effect test results in Table 10 confirm that the impact of artificial intelligence on industrial chain resilience exhibits significant variation depending on the level of data element development. When data element development falls below the threshold value (T < 0.374), AI still exerts a significant positive effect on industrial chain resilience (coefficient: 0.554), albeit relatively limited. Once this threshold is crossed (T ≥ 0.374), the impact of AI significantly intensifies (coefficient: 1.104). This indicates that during phases characterised by weak data foundations and inefficient circulation, the technological potential of AI is constrained. Conversely, once data elements accumulate to a certain level, forming an ecosystem of efficient sharing and compliant application, AI can more profoundly empower supply chain coordination, risk early warning, and innovation iteration, thereby substantially enhancing the overall resilience of industrial chains. This finding suggests that advancing AI integration with manufacturing must not solely focus on technology itself. It is imperative to concurrently cultivate data element markets and refine data governance systems. By overcoming the ‘data threshold,’ the transformative potential of AI within industries can be fully unleashed, fostering a mutually reinforcing development paradigm where ‘data-driven’ and ‘AI-empowered’ approaches complement each other.

**Table 10:** Threshold effect.

	(1)
	Resi
*AI*(*T* < 0.374)	0.554***
	(3.26)
*AI*(*T* ≥ 0.374)	1.104***
	(6.51)
*URB_inc*	0.000**
	(2.60)
*R&D*	0.002***
	(13.52)
*TAX*	3.894
	(1.29)
*Ind_third*	-0.021**
	(-2.05)
*_cons*	-6.981***
	(-14.78)
N	360
R^2^	0.859

## Further analysis

### Regional heterogeneity

**Fig. 2:**
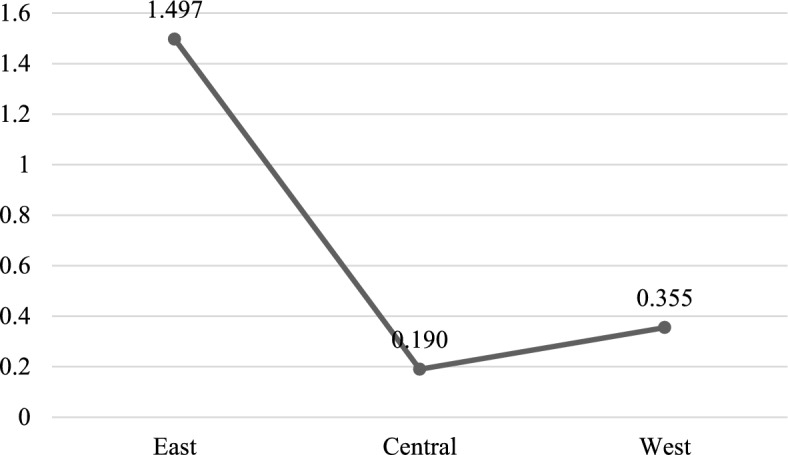
Regional coefficient.

To gain a more precise understanding of the boundaries and applicability of artificial intelligence in manufacturing transformation, and to reveal the differentiated manifestations of its impact effects across distinct regional economic ecosystems, this study conducted a regionally disaggregated regression analysis based on the National Bureau of Statistics’ classification standards for eastern, central, and western regions. This regional comparative perspective facilitates the spatial identification of key contextual factors influencing technological empowerment outcomes, thereby providing empirical foundations for tailored regional industrial intelligence policies. As shown in (Table 11), the impact of artificial intelligence exhibits pronounced regional heterogeneity, highlighting the systemic moderating effects of regional resource endowments, industrial foundations, and technological application environments. At the same time, the coefficients were visualized, as shown in (Fig. 2).

**Table 11:** Subregional heterogeneity test.

	(1)	(2)	(3)
	East	Central	West
*AI*	1.497***	0.190	0.355**
	(4.18)	(0.65)	(2.06)
*URB_inc*	0.000***	0.000***	0.000***
	(5.08)	(10.71)	(6.39)
*R&D*	0.004***	0.001***	0.001***
	(12.34)	(3.80)	(5.51)
*TAX*	-8.637	-14.991***	3.527
	(-1.23)	(-2.74)	(0.88)
*Ind_third*	-0.005	-0.001	-0.014
	(-0.10)	(-0.07)	(-1.04)
_cons	-13.116***	-8.482***	-9.817***
	(-5.07)	(-8.89)	(-11.31)
N	132	96	120
R^2^	0.912	0.962	0.887

#### Eastern region: significant and robust positive effects

Within the eastern region sample, the estimated coefficient for artificial intelligence is 1.497, which is statistically significant at the 1% level. This indicates a substantial positive effect of AI on enhancing industrial chain resilience. This pronounced impact can be attributed to the region’s robust digital infrastructure, high concentration of innovative resources, and relatively advanced level of industrial intelligence. Well-developed 5G networks, data centers, and widespread enterprise digital adoption provide a solid foundation for the implementation and deep integration of AI technologies. Furthermore, active capital markets, abundant high-skilled talent pools, and mature industry-university-research collaboration mechanisms collectively facilitate the effective integration of AI across manufacturing processes, including R&D, production, and management. Consequently, AI plays a pivotal role in improving industrial chain responsiveness, optimizing coordination within supply chains, and strengthening overall resilience.

#### Western regions: significant yet suboptimal effects

In the western regions, the coefficient for AI is 0.355, which is significantly positive at the 5% level. This indicates that the technology-empowering effect has begun to materialize, although its magnitude remains considerably weaker than in the eastern region. This disparity may stem from factors such as relatively lagging digital infrastructure, a still-developing application ecosystem, and an insufficient supply of localized AI solutions. Nevertheless, driven by national strategies like the East–West Computing Resource Transfer Initiative, the western region is actively leveraging its advantages in energy costs and land availability to deploy computing infrastructure. This lays a foundation for future AI applications. The currently weaker effect also suggests that, beyond infrastructure development, the western region must simultaneously strengthen technology introduction and assimilation, foster localized scenario innovation, and cultivate interdisciplinary talent. These efforts are crucial to shortening the transition from “technological availability” to “effective industrial empowerment.”

#### Central regions: effects remain insignificant amid structural constraints

Notably, for the central region sample, the coefficient on artificial intelligence is 0.190 and statistically insignificant. This suggests that AI’s positive effect on the resilience of local manufacturing chains has not yet been systematically realized. A plausible explanation is that, as a traditional manufacturing base, the central region faces dual structural constraints while pursuing industrial upgrading. On one hand, its digital infrastructure, technological reserves, and ability to attract high-end talent lag behind those of the eastern region. On the other hand, during industrial transfer, it often concentrates on manufacturing segments while lacking capabilities in higher-value chain activities—such as R&D, design, and brand marketing—as well as in full-chain digital collaboration. Consequently, AI applications may remain limited to localized automation substitution, failing to deeply integrate into organizational transformation and systemic resilience building across the industrial chain. This could explain the absence of a tangible resilience-enhancing effect.

### Quantile heterogeneity analysis

**Fig. 3:**
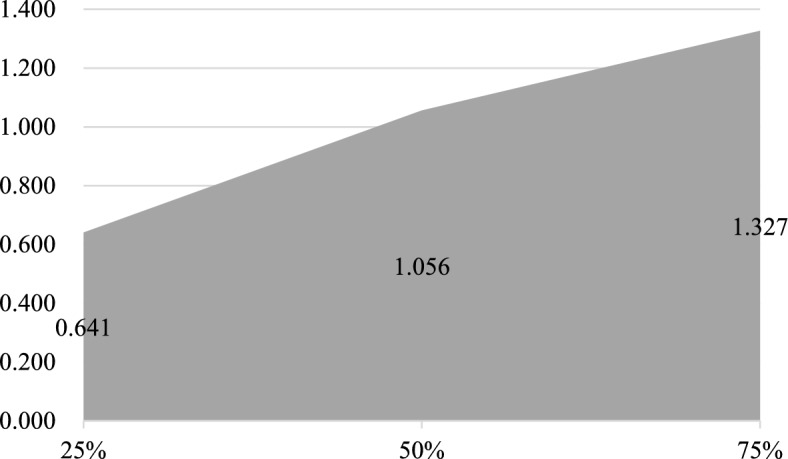
Quantile coefficients.

To examine whether the impact of artificial intelligence on manufacturing industrial chain resilience varies across different resilience levels, and to address the limitation that traditional mean regression may obscure heterogeneity across the conditional distribution, this study employs quantile regression. This method effectively captures the differential effects of the explanatory variable at various points of the dependent variable’s distribution. It is particularly suited for analyzing the role of AI in manufacturing supply chains with differing resilience levels, thereby offering a more nuanced understanding of their relationship. Three representative quantiles—25, 50, and 75%—were selected for analysis. The empirical results are presented in (Table 12). At the same time, the coefficients were visualized, as shown in (Fig. 3).

**Table 12:** Quartile regression results.

	(1)	(2)	(3)
	25%	50%	75%
*AI*	0.641***	1.056***	1.327***
	(3.31)	(4.10)	(4.45)
*URB_inc*	0.000***	0.000***	0.000***
	(7.40)	(3.46)	(3.52)
*R&D*	0.002***	0.002***	0.002***
	(12.62)	(9.32)	(7.91)
*TAX*	7.658**	2.320	0.636
	(2.09)	(0.48)	(0.11)
*Ind_third*	0.010	0.029	0.041*
	(0.75)	(1.60)	(1.97)
_cons	-10.126***	-9.727***	-11.081***
	(-7.62)	(-5.52)	(-5.42)
N	360	360	360

Table 12 clearly demonstrates that AI’s impact on manufacturing supply chain resilience exhibits a pronounced quantile-increasing pattern. Specifically, at the 25th percentile (low resilience) of the supply chain resilience distribution, AI’s coefficient is 0.641, significant at the 1% level; As resilience levels rise to the median (50th percentile), the impact coefficient increases to 1.056, maintaining its significance; at the high 75th percentile, the coefficient further rises to 1.327, retaining high significance. This systematic trend indicates that AI’s enabling effect is not homogeneous but progressively strengthens as the baseline resilience of the industrial chain increases.

The aforementioned pattern of ‘increasing marginal effects’ reveals that the underlying mechanism through which AI influences industrial chain resilience exhibits pronounced path dependency and systemic synergistic effects. Within industrial chains characterised by lower resilience levels (corresponding to lower quantiles), systems often face multiple constraints such as weak foundations, low digitalisation levels, and rigid organisational processes. At this stage, AI applications are predominantly concentrated on automating or informatising specific segments. While this yields some efficiency gains and risk buffering, the overall resilience-enhancing effect remains relatively modest due to insufficient system-wide coordination and limited absorption capacity. Conversely, when industrial chains possess a robust resilience foundation (corresponding to high quantiles), the system typically exhibits a more sophisticated supply chain network, agile organisational structures, extensive data accumulation, and strong technological assimilation capabilities. This creates an ideal application environment and synergistic conditions for AI to perform advanced functions—such as intelligent forecasting and dynamic scheduling based on full-chain data, cross-process resource optimisation, and adaptive production process reconfiguration. Highly resilient systems are better positioned to establish a positive feedback loop with AI technologies: ‘technology empowerment → enhanced resilience → further optimisation of the technological application foundation’. This significantly amplifies the marginal contribution of AI, manifesting a Matthew effect where the strong grow stronger.

## Conclusions and implications

### Research findings

This study employs panel data from 30 Chinese provinces spanning 2012 to 2023 to systematically examine the impact mechanisms, nonlinear characteristics, and spatial heterogeneity of artificial intelligence (AI) on the resilience of manufacturing industrial chains. The analysis, conducted across multiple dimensions including technological empowerment, systemic coordination, and contextual moderation, leads to the following principal conclusions:

First, artificial intelligence exerts a significant and robust direct positive effect on the resilience of manufacturing industrial chains. Benchmark regressions, along with a series of endogeneity and robustness tests, demonstrate that AI development effectively enhances an industrial chain’s capacity to withstand, recover from, and adapt to internal and external shocks, thereby validating Hypothesis H1.

Second, artificial intelligence not only directly enhances industrial chain resilience but also generates an indirect positive effect by stimulating regional economic development. Mediating effect tests confirm that AI, by driving regional economic growth, fosters a more favorable macroeconomic environment and industrial ecosystem, which in turn supports greater resilience. This establishes a transmission pathway of “technology promotes growth, and growth underpins resilience,” supporting Hypothesis H2.

Third, the urbanization process exerts a significant positive moderating effect on the relationship between AI and industrial chain resilience. The moderation analysis indicates that higher urbanization levels amplify AI’s resilience-enhancing impact. This suggests that the factor agglomeration, improved infrastructure, and optimized institutional environment associated with urbanization provide crucial support for the implementation and collaborative application of AI technologies, validating Hypothesis H3.

Fourth, the impact of AI on industrial chain resilience exhibits a nonlinear threshold characteristic dependent on the development level of data elements. Threshold effect tests identify a single threshold: once the development level of data elements surpasses this threshold, the positive effect of AI intensifies significantly, demonstrating a pattern of “increasing marginal returns.” This indicates that data elements serve as a foundational condition for AI’s enabling effects. When the data ecosystem is underdeveloped, the technological potential of AI remains constrained, confirming Hypothesis H4.

Fifth, the enabling effects of AI exhibit pronounced regional and conditional heterogeneity. Regional regressions reveal a spatial pattern in which the strength of AI’s impact follows a hierarchy of “strongest in the East, followed by the West, and least pronounced in the Central region,” reflecting the systemic moderating roles of regional digital infrastructure, industrial structure, and institutional environment. Quantile regression further indicates that AI’s positive effect intensifies with higher levels of existing industrial chain resilience, exhibiting path-dependent characteristics of “the strong growing stronger” through synergistic reinforcement.

### Policy implications

Based on the above findings, the following policy implications are proposed to advance AI’s deep empowerment of manufacturing industrial chain resilience and achieve coordinated regional high-quality development:

First, implement a tiered AI empowerment strategy with differentiated guidance. For highly resilient eastern regions, focus should be placed on the deep integration of AI within industrial chain coordination, intelligent decision-making, and ecological innovation, establishing ‘AI + Resilience’ demonstration zones and pioneer chains. In central and western regions, equal emphasis must be placed on ‘hard investments’ in digital infrastructure and ‘soft cultivation’ of technological application ecosystems, particularly strengthening support in data governance, localised scenario innovation, and cross-disciplinary talent development to shorten technology penetration cycles.

Second, establish a tripartite synergistic support system encompassing technology, economics, and institutions. While advancing AI R&D and industrial applications, ensure effective alignment with regional economic development strategies and new urbanization initiatives. Strengthen institutional safeguards including digital infrastructure, data element market cultivation, standardisation, and security governance to foster a multi-tiered policy environment conducive to unlocking AI’s systemic enabling effects.

Third, develop robust dynamic monitoring and assessment mechanisms for industrial chain resilience. It is recommended to develop regional and sector-specific diagnostic frameworks for industrial chain resilience, identifying shortcomings and bottlenecks across varying resilience levels. For low-resilience segments and regions, policies should prioritise digitalisation adoption and foundational capability enhancement. Conversely, highly resilient systems should be encouraged to adopt advanced applications such as predictive maintenance and intelligent scheduling, thereby continuously strengthening their capacity to withstand systemic risks and structural transformations.

Fourth, promote cross-regional coordination and experience sharing to narrow the resilience gap. Encourage the establishment of mechanisms for East–West regional collaboration on smart manufacturing platforms, industrial data sharing, and algorithmic model cooperation. Through joint industrial park development, talent exchanges, and case study dissemination, facilitate the replication and diffusion of effective AI-enabled resilience models across regions, fostering an open, collaborative, and mutually reinforcing development framework.

### Research limitations and future prospects

While this study reveals, based on macro-level provincial data, the positive impact of artificial intelligence (AI) on the resilience of manufacturing industrial chains and its underlying transmission mechanisms, the following areas merit further exploration.

On the one hand, data granularity and methodological approaches require deepening. The current research primarily relies on provincial panel data, which, while capable of capturing overall regional trends, falls short of delineating the heterogeneous characteristics of micro-level firm behavior and their dynamic roles in shaping resilience. Future research could consider acquiring more granular firm-level data and incorporating machine learning algorithms, such as random forests or gradient boosting models, to address the potentially complex nonlinear relationships and interaction effects between AI and industrial chain resilience. This would allow for a more precise revelation of the intrinsic dynamic mechanisms of technological empowerment. However, this path will inevitably confront practical challenges related to data availability, privacy protection, and computational resources.

On the other hand, the translation into industrial practice and implementation pathways warrants further investigation. Although the research findings possess statistical significance, in practice, the empowering effect of AI is influenced by multiple factors such as industry characteristics, firm size, and organizational culture. Its practical promotion still faces prominent obstacles including high transformation costs, data silos, and talent shortages. Therefore, future work could integrate typical case studies and in-depth field research to analyze differentiated application scenarios and bottlenecks across various manufacturing subsectors. Furthermore, by launching pilot projects on "AI + Industrial Chain Resilience" and establishing collaborative "government-industry-academia-research-application" platforms, academic insights can be transformed into actionable industry solutions, thereby effectively translating theoretical discoveries into practical applications.

## Data Availability

All data used in this study, covering 30 provinces (autonomous regions and municipalities directly under the Central Government) of China from 2012 to 2023, were extracted from the *China Statistical Yearbook* and the corresponding local statistical yearbooks of each province (autonomous region and municipality directly under the Central Government). All these yearbooks are official and publicly available publications. The data can be retrieved from the official website of the National Bureau of Statistics of the People’s Republic of China (http://www.stats.gov.cn/) and the official platforms of statistical bureaus of respective provinces (autonomous regions and municipalities directly under the Central Government). The model codes and preprocessed datasets employed in this study are available from the corresponding author upon reasonable request.

## References

[1] Xinrong, Mu. & Hui, G. Mechanisms and pathways through which digital trade development empowers industrial chain resilience. *Contemp. Econ.* **42** (12), 16–31 (2025).

[2] Shitong, Z., Xiaodan, W. & Yutang, S. The impact of establishing artificial intelligence innovation application pilot zones on enhancing industrial chain resilience: an empirical study based on dual machine learning. *Sci. Technol. Progress Policy* **42** (18), 1–9 (2025).

[3] Pan, H., Gu, H., Ye, L. The impact of artificial intelligence industry availability on innovation quality in manufacturing enterprises [J/OL]. *Sci. Technol. Manag.* 1-17 https://link.cnki.net/urlid/11.1567.G3.20251212.1314.002.

[4] Xiaoyong, Q. et al. Artificial intelligence application, high-quality development of manufacturing export enterprises, and production network spillovers. *J. Beijing Inst. Technol. (Soc. Sci. Ed.)* **27** (06), 149–165 (2025).

[5] Yan, W. & Jingshuang, Li. Artificial intelligence empowering energy economic resilience: An examination of mechanisms and spatial spillover effects. *Indust. Technol. Econ.* **44** (10), 128–137 (2025).

[6] Davies, S. Pandemics and the consequences of COVID-19. *Econ. Aff.* **40** (2), 131–137 (2020).

[7] Xingzhi, X. & Shaolin, Li. Industrial chain resilience amidst great transformation: Generative logic. *Pract. Concerns Policy Orientat. Reform.* **11**, 1–14 (2022).

[8] Yuxin, Li. & Xiaoying, J. The impact of digital-physical integration on the resilience of manufacturing industrial chains: An Analysis based on the dual ‘technology-data’ drivers of enterprises. *China Circul. Econ.* **39** (08), 3–18. 10.14089/j.cnki.cn11-3664/f.2025.08.001 (2025).

[9] Xiaolong, Li., Ziyi, Z. & Qiang, Z. Technological financial policies, innovation factor agglomeration and industrial chain resilience: Empirical evidence from a quasi-natural experiment. *Bus. Res.* **03**, 1–9. 10.13902/j.cnki.syyj.2025.03.002 (2025).

[10] Kozma, D. & Varga, P. Supporting digital supply chains by IoT frameworks: Collaboration, control, combination. *Infocommun. J.* **12** (4), 22–32 (2020).

[11] Tan, W. J., Cai, W. & Zhang, A. N. Structural-aware simulation analysis of supply chain resilience. *Int. J. Prod. Res.* **58** (17), 5175–5195 (2020).

[12] Ivanov, D. Revealing interfaces of supply chain resilience and sustainability: a simulation study. *Int. J. Prod. Res.* **56** (10), 3507–3523 (2018).

[13] Qiguang, An. et al. Digital-physical integration and industrial chain resilience: impact effects and operational mechanisms. *Stat. Decis. Making* **41** (22), 61–67. 10.13546/j.cnki.tjyjc.2025.22.010 (2025).

[14] Teixeira, A. R., Ferreira, J. V. & Ramos, A. L. Intelligent supply chain management: A systematic literature review on artificial intelligence contributions. *Information* **16** (5), 399 (2025).

[15] Götz, M. Cluster role in industry 4.0–a pilot study from Germany. *Compet. Rev. Int. Business J.* **31** (1), 54–82 (2021).

[16] Buchmeister, B., Palcic, I. & Ojstersek, R. Artificial intelligence in manufacturing companies and broader: An overview. *DAAAM Int. Sci. Book* **1** (18), 81–98 (2019).

[17] Yun Jae-in. Comparative Analysis of the US-Japan and US-China Semiconductor Disputes (Doctoral dissertation, Seoul National University Graduate School).

[18] Yuanyuan, L. Artificial intelligence, innovation factor allocation and industrial chain resilience. *Stat. Decis. Making* **41** (18), 117–122 (2025).

[19] Fangyi, J. & Jiayue, L. Artificial intelligence, digital-physical technology convergence and resilience of manufacturing industrial chains. *J. Yunnan Minzu Univ. (Philos. Soc. Sci. Ed.)* **42** (06), 86–99 (2025).

[20] Bin, Wu., Shuangwei, B. & Xin, Z. Application of industrial chain resilience in evaluating artificial intelligence industrial chains. *Res. Sci. Technol. Manag.* **43** (07), 199–204 (2023).

[21] Qiao, P., Du, X., Han, X. How can generative artificial intelligence enhance the resilience of manufacturing enterprises? [J/OL]. *Sci. Sci. Technol. Manag.* 1–22 (2025).

[22] Huiru, C. & Yunhong, H. The impact of artificial intelligence on total factor productivity in manufacturing enterprises. *J. Nanjing Univ. Finance Econ.* **03**, 46–56 (2025).

[23] Lin, T. & Shijie, Z. Measuring virtual agglomeration levels, spatial disparities and convergence in China. *J. Zhejiang Univ. (Human. Soc. Sci. Ed.)* **53** (07), 75–97 (2023).

[24] Yonggang, Li. & Yue, L. Does land-based investment attraction enhance urban economic resilience? Empirical evidence from micro-level land transaction data. *J. Huazhong Univ. Sci. Technol. (Soc. Sci. Ed.)* **39** (04), 69–81 (2025).

[25] Huamin, H. & Ling, L. Measuring the level, regional disparities and evolutionary trends of new quality productivity in Western China’s agriculture. *Contemp. R. Finan. Econ.* **09**, 2–9 (2025).

[26] Xin, S. Artificial intelligence technological innovation and green low-carbon development in agriculture: Perspectives from new quality productivity and ecological product value realisation. *Res. Technol. Econ. Manag.* **11**, 134–141 (2025).

[27] Changnan, Wu. The ‘Jinjiang experience’: Exploring the integrated development of new industrialisation and urbanization. *Fujian Forum (Human. Soc.Sci. Ed.)* **10**, 132–140 (2022).

[28] Hui, S., Xie, Y., Wang, Z. Study on the impact of market-oriented allocation of data factors on the integration of innovation chains and industrial chains in high-tech industries [J/OL]. *Soft Sci.* 1–15 (2025).

[29] Xinxin, L. & Xuanxin, H. Artificial intelligence and manufacturing resilience: Internal mechanisms and empirical tests. *Econ. Manag.* **45** (11), 48–67 (2023).

[30] Xianping, S. & Yuchi, C. How can artificial intelligence empower the ascent of manufacturing in global value chains during the 15th five-year plan period? — An analysis based on the technology-economy-society paradigm. *Acad. Forum* **48** (3), 99–112 (2025).

[31] Xuecheng, Y., Jing, G. & Dongxiao, Y. Research on the transmission pathways of technological progress in artificial intelligence on employment structure in high-tech manufacturing industries. *J. Beijing Univ. Technol. (Soc. Sci. Ed.)* **24** (2), 110–123 (2024).

[32] Zeng, C., Wei, X. & Yu, L. How generative artificial intelligence reshapes labour markets: A review and future outlook. *Ind. Econ. Rev.* **3**, 82–102 (2025).

